# Accelerated dual-contrast quantitative first-pass perfusion MRI of the mouse heart with compressed sensing

**DOI:** 10.1186/1532-429X-15-S1-W17

**Published:** 2013-01-30

**Authors:** NK Naresh, X Chen, P Antkowiak, Y Xu, BA French, FH Epstein

**Affiliations:** 1 Biomedical Engineering, University of Virginia, Charlottesville, VA, USA; 2 Radiology, University of Virginia, Charlottesville, VA, USA

## Background

Myocardial blood flow (MBF) imaging in gene-modified mice may be used to elucidate molecular mechanisms that underlie coronary vascular function and dysfunction. First pass Gd-enhanced MRI is well-established in humans and has been recently investigated in mice[1-3]. The rapid heart rate and small size of the heart present challenges to performing quantitative first pass imaging in mice. A dual contrast acquisition simplifies the procedure (one Gd injection) and may provide more accurate results because the arterial input function (AIF) and tissue function (TF) are acquired under identical conditions. We developed a compressed sensing (CS)-accelerated first pass sequence for mice with a dual contrast acquisition.

## Methods

MRI was performed on a 7T system (Bruker, Germany). C57Bl/6 mice were anesthetized with 1.25% isoflurane and maintained at 36±0.5^0^C during MRI. Mice were imaged at rest (n=6), with the vasodilator ATL313 (25μg/kg) (n=6), and on day 1 post-myocardial infarction (MI) (n=6). A dual contrast saturation-recovery sequence with k_y_ and time domain undersampling was used to acquire first-pass Gd-enhanced images. Two slices were acquired within a cardiac cycle, one to obtain the AIF and the other to obtain the TF. For both slices, the center (50%) of k-space was acquired at the Nyquist rate while higher spatial frequencies were randomly undersampled. The acceleration factors for the AIF and TF slices were 6 and 4, respectively. Other imaging parameters were: TE/TR=1.2/2.1ms, FOV=25.6x18-20.8mm^2^, resolution=200μm^2^, matrix=128x74-104, alpha=15^0^, slice thickness=1mm, saturation delay=15/70ms for AIF/TF. A motion-compensated CS algorithm was used to reconstruct the undersampled images[4]. Fermi function deconvolution quantified perfusion[5] in the entire slice (Baseline and ATL313) or in the infarct and remote zones (Day 1 post-MI), where late gadolinium enhancement (LGE) defined the infarct zone.

## Results

**Figure 1:**
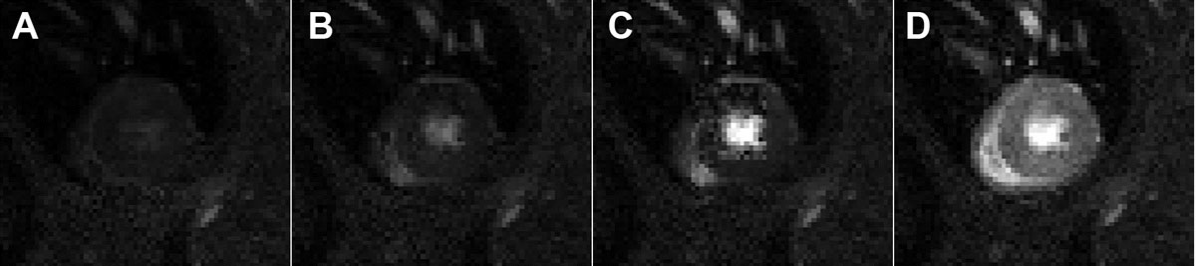
Example first-pass images obtained from a mouse at rest prior to injection (A), 0.5 s post-injection (B), 1 s post-injection (C) and 5 s post-injection of Magnevist (Gd-DTPA) (D).

Figure 1 shows example first-pass images obtained from a mouse at rest. For normal mice, mean baseline perfusion was 5.3±2.1 ml/g/min and it increased to 11.8±1.5 ml/g/min with ATL313 (p<0.05 vs. rest). Perfusion reserve was 2.5±0.9. Mean perfusion on day 1 post-MI was 2.2±0.6 ml/g/min (p<0.05 vs. rest) in the infarct zone, and 5.5±1.5 ml/g/min in the remote zone.

**Figure 2:**
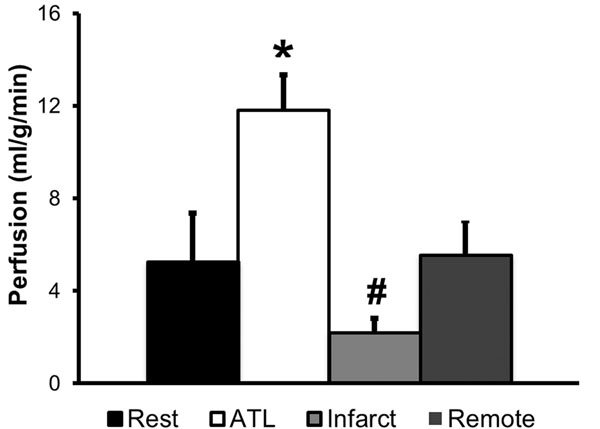
Baseline perfusion was found to be 5.3±2.1 ml/g/min and it increased to 11.8±1.5 ml/g/min with ATL313 (p<0.05 vs. rest). Perfusion on day 1 post-MI was found to be 2.2±0.6 ml/g/min in the infarct zone (p<0.05 vs. rest) and 5.5±1.5 ml/g/min in the remote zone.

## Conclusions

Accelerated first-pass MRI using undersampling and CS enables the implementation of a dual-contrast method, which can quantify perfusion in the mouse heart over a wide range of MBF. MBF reserve can also be measured. These methods promise to prove useful in the assessment of therapies for MI, and may shed light on key molecular mechanisms that underlie coronary vascular dysfunction.

## Funding

This work was funded in part by AHA Predoctoral Award 11PRE7440117, NIH R01 EB001763 and US-Israel Binational Science Foundation grant 2007290.
